# Load-deformation behaviour of weft-knitted textile reinforced concrete in uniaxial tension

**DOI:** 10.1617/s11527-021-01797-5

**Published:** 2021-11-04

**Authors:** Minu Lee, Jaime Mata-Falcón, Walter Kaufmann

**Affiliations:** Institute of Structural Engineering (IBK), Swiss Federal Institute of Technology (ETH Zürich), Stefano-Franscini-Platz 5, HIL E 36.2, 8093 Zürich, Switzerland

**Keywords:** Textile reinforced concrete, Experimental study, Uniaxial tension, Cracking behaviour, Weft-knitted textile, KnitCrete

## Abstract

Weft-knitted textiles offer many advantages over conventional woven fabrics since they allow the fabrication of doubly curved geometries without the need of stitching multiple patches together. This study investigated the use of high-strength continuous fibres as knitted textile reinforcement, focusing on various knitting patterns, fibre materials, coating types and spatial features to enhance the bond conditions between concrete and reinforcement. The bond is of particular interest since the contact surface of knitted textiles is fundamentally different due to their closed surface, compared to commercially available textile reinforcement, which is normally formed as orthogonally woven grids of rovings. An experimental campaign consisting of 28 textile-concrete composites was conducted, where digital image correlation-based measurements were used to assess the load-deformation behaviour and to analyse the crack kinematics. The results showed a beneficial post-cracking behaviour for epoxy coated configurations with straight inlays. The comparison of these configurations with conventional textile reinforcement generally showed a similar behaviour, but with higher utilisation compared to the filament strength. The Tension Chord Model, which assumes a constant bond stress-slip relationship, was adapted for the specific geometry of the knitted reinforcement, and it was used for the estimation of bond stresses and a post-diction of the experimental results, generally showing a good agreement.

## Introduction

As the construction industry strives to reduce its ecological footprint, textile reinforced concrete has been identified as a solution with high potential to meet the demands for more efficient and lightweight structures with less material consumption. Textile reinforcement is typically made of continuous high-strength multifilament yarns that form bundles of thicker strands (rovings) [1]. The use of materials not affected by corrosion such as aramid, carbon or glass fibre enable the construction of much thinner elements compared to conventional steel reinforcement since there is no need for a thick concrete cover to prevent corrosion [2]. The coating or even full impregnation of the rovings with a resin (e.g. epoxy) improves their resistance against abrasion and the inter-fibre friction [3], which enhances their robustness against lateral loading (local lateral pressure at the crack edges of textile reinforced concrete composites can damage the fibres, reducing the tensile strength of the roving [4]) and leads to a more homogeneous stress distribution within the cross section of a single strand [5]. The high tensile strength of the fibres (around 3000 MPa–4000 MPa) allows an efficient use of materials in ultimate limit state. The low ratio of stiffness (glass fibre: ca. 70 GPa, carbon fibre: ca. 240 GPa) to strength (when compared to typical reinforcing steel with a Young’s modulus of ca. 205 GPa and a yield strength of ca. 500 MPa) leads to large elastic deformations at failure, which is generally favourable since it announces an upcoming rupture. The failure mode itself is brittle since these fibrous materials normally lack ductility. However, serviceability requirements might become governing for the final design.

Although textile reinforcements exist in a wide variety of geometries with even spatial arrangements being possible [6], the vast majority of applications for both new structures and strengthening of existing structures uses flat sheets of orthogonally woven grids of straight rovings [7, 8]. While this type of reinforcement is well suited for flat and single curved elements [9, 10], there are great challenges remaining for doubly curved surfaces since the flat reinforcement has to be cut in many patches to fit the complex geometry. This creates some problems with directionality of the reinforcement grid, and leads to many unwanted lap splices, which cause some uncertainty for the continuous action of the reinforcement and reduce material efficiency.

Weft-knitted textiles offer great advantages in the geometric definition with respect to woven textiles. CNC knitting machines allow the local variation of length and width of the textile creating doubly curved surfaces and enable spatial features such as channels or ribs [11] as well as the integration of straight yarns in both warp and weft direction within the knitted fabric [8]. The possibilities and advantages of using knitted textiles in construction have recently been explored and are being showcased in the KnitCrete technology [12] developed at ETH Zürich, which is a stay-in-place formwork system: the textile is tensioned in a scaffolding frame or supported by elements such as bending-active rods or inflatables. It is initially coated with a thin layer of fast-setting high strength cement paste, which — after hardening — serves as stable continuous formwork for casting the concrete. However, although the technology has already been applied to large construction projects such as the KnitCandela pavilion in Mexico City [13], the integration of reinforcement in the fabrication process has still not been solved. The authors addressed potential approaches of reinforcing complex concrete structures using integrated flexible formwork in [14]. A promising solution is the direct use of the stay-in-place formwork as weft-knitted textile reinforcement by utilising high-strength fibrous materials as knitting yarn, which can considerably simplify the construction sequences by combining the erection of formwork and the placing of reinforcement into a single fabrication step.

**Fig. 1:**
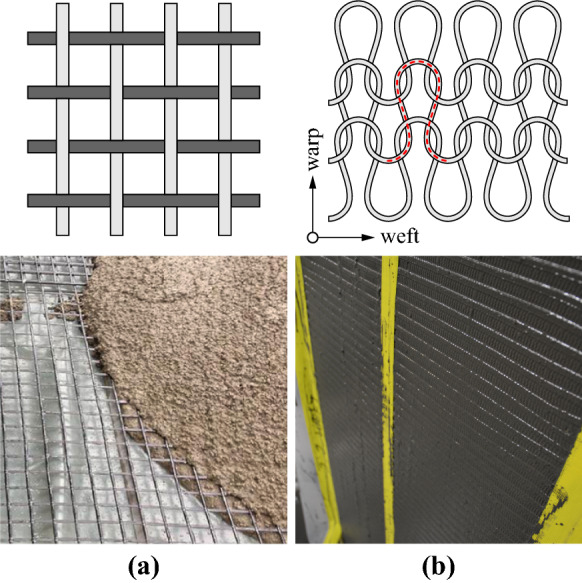
Textile reinforced concrete: **a** conventional woven grid of straight rovings within concrete matrix (image source: solidian GmbH); **b** knitted loop structure and closed surface of a weft-knitted textile with cement paste coating

While the structural behaviour of conventional textile reinforcement has been studied in detail (e.g. [4, 15–20]), knowledge of weft-knitted textile reinforced concrete is still scarce and there is only little existing literature (e.g. [21, 22]). Several studies on the bond behaviour of uncoated and coated textiles indicate the pronounced influence of the coating type and yarn texture on the bond conditions [23–27]. The behaviour of concrete structures reinforced with knitted textiles is presumably significantly different from that of conventional woven textile reinforced concrete due to the fundamentally different bond conditions: knitted textiles typically present a closed surface after coating due to the small loop size, while in conventional textile reinforcement the concrete normally flows through the textile (see Fig. 1). Furthermore, the textiles in this study make use of a manual process that is easily applicable on construction site but might exhibit more local variation of coating quality, while conventional textile reinforcement is produced in controlled industrial conditions where the full impregnation of the yarns can be ensured (e.g. by vacuum impregnation).

This study addresses the structural behaviour of weft-knitted textile reinforced concrete. To this end, an experimental study by means of uniaxial tension tests on thin concrete elements with weft-knitted textile reinforcement was conducted. The investigations focused on various knitting patterns, fibre materials, types of coating and spatial features within the textile to enhance the bond at the interface between concrete and reinforcement, which considerably influences the post-cracking behaviour and deformation capacity. Digital image correlation-based measurements were used to assess the deformations, cracking pattern and crack kinematics. The experimental data allowed the back-calculation of bond stresses using the Tension Chord Model [28]. Furthermore, the performance of the fibres is compared to experimental data of conventional textile reinforcement from existing literature, and their potential use in structural applications is discussed.

The study, therefore, presents fundamental results and findings on the structural behaviour of a newly developed reinforcement type made of continuous high-strength fibrous materials, which has not yet been documented. For the actual application in planar and spatial structural elements, further investigations on more complex loading conditions such as biaxial membrane forces, bending moments or in-plane shear are needed.

## Experimental programme

The experimental study presented in this article consisted of 28 specimens that were manufactured and tested to failure in the structures laboratory at ETH Zürich. The specimen layout and test setup followed on the recommendations of RILEM TC 232-TDT [29] for the testing of textile reinforced concrete in uniaxial tension, with some minor adjustments as outlined in the following sections. Additionally, strength and stiffness of the fibres were determined with direct tension tests on straight yarns with and without epoxy coating.

### Materials and manufacturing of specimens

The dimensions of the specimens were 800 mm in length and 200 mm in width. Compared to the RILEM recommendations (which give a specimen width of 60 mm), the specimens were wider to minimise edge effects that arise from the lateral contraction of knitted textiles loaded in uniaxial tension. The thickness of the specimens was between 12 and 16 mm, where the coated textile was centred in the cross section with equal concrete cover on both sides. The experimental program containing single roving tests and experiments performed on the composites is summarised in Tables 1 and 2. For each configuration, two specimens were tested. All components used for the production of the textile reinforced concrete tension ties were manufactured from commercially available base materials, which are described in the following sections.

**Table 1:**
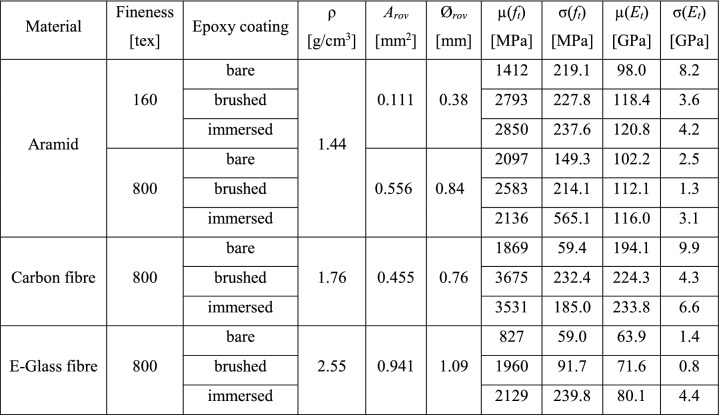
Mechanical characterisation of the fibre materials: mean ($\mu$) and standard deviation ($\sigma$) of tensile strength ($f_t$) and Young’s modulus ($E_t$) obtained from single roving tests

**Table 2:**
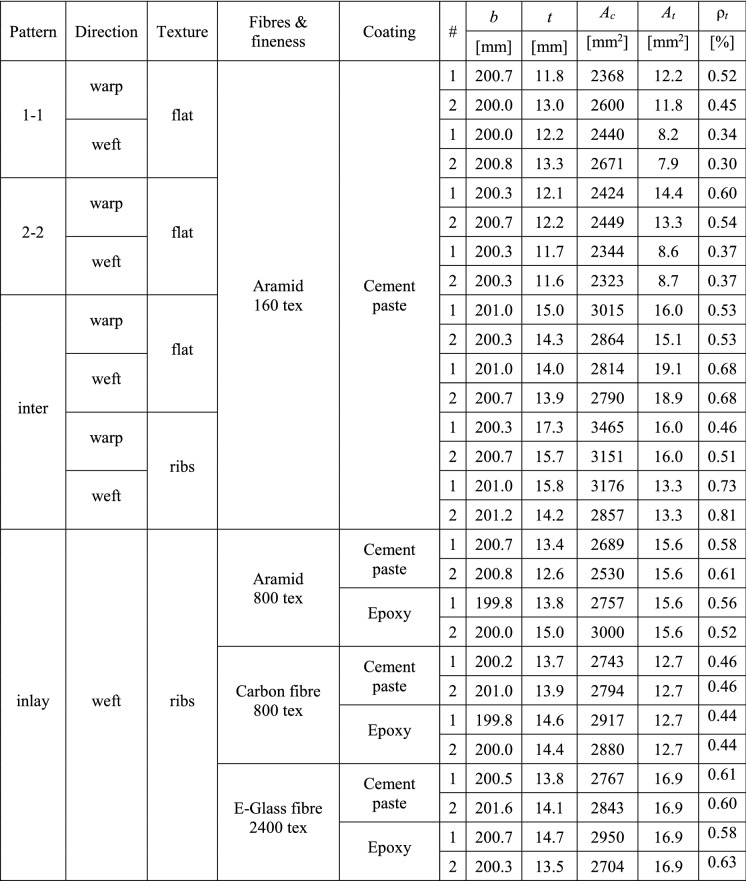
Configurations, dimensions and reinforcement content of tested tension ties

#### Textile reinforcement

The study covered three different materials of fibres for the textile reinforcement: aramid (AF), carbon (CF) and E-glass (GF) fibres. Generally, the use of E-glass in cementitious composites should be avoided due to its poor durability in alkaline environments [1]. However, this material was used due to a delivery shortage of direct rovings of alkali-resistant (AR) glass fibres. The short-term mechanical properties of E-glass and AR-glass fibres are very similar [30] and therefore, the general findings of this study apply to AR fibres as well.

**Fig. 2:**
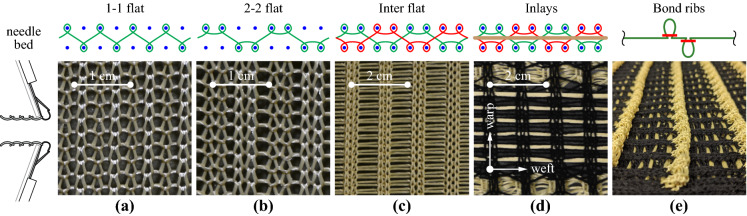
Knitting patterns: single layer **a** 1–1, **b** 2–2; double layer **c** interlock; **d** straight inlays within non-structural base textile; **e** additional bond ribs used in some specimens

The textile reinforcement was manufactured with a CNC double bed knitting machine (Steiger Libra 3.130), enabling a wide spectrum of knitting patterns. Depending on the sequence of activated needles in a double bed knitting machine, single and double layer textiles are possible and connections along the fabric can create spatial features such as channels and ribs [11, 14]. The various knitting patterns differ in roving density, stretchiness, textural roughness, and other properties. Figure 2 shows an overview of the knitting patterns used in the study and their schematic generation as sequence of loops formed on either side of the double needle bed.

The first two patterns in Fig. 2a and b were single layer fabrics that alternate between the needle beds. The interlocked textile in Fig. 2c mirrors itself with every new row creating a double-layer textile. These knitting patterns were limited to the use of aramid yarns with relatively low fineness (160 tex; 1 tex = 1 g per km of yarn): the hooks of the knitting needles are not able to grab thicker yarns, and carbon or glass fibre yarns would break during fabrication with such small bending radii. These types of textiles will be referred to as ‘directly knitted reinforcement’. They were tested in both warp and weft direction (parallel and perpendicular to the direction of the knitting process).

The knitting machine also allows for the integration of straight rovings (called inlays), which are guided within the knitted textile (thicker yellow yarns in Fig. 2d). This significantly increases the stiffness and strength of the reinforcement since it introduces a more specific directionality to the textile matching the type of loading and allows the use of thicker yarns. Aramid and carbon fibre rovings with a fineness of 800 tex at an average spacing between the yarns of 7.1 mm and glass fibre rovings with a fineness of 2400 tex at an average spacing of 11.1 mm were used as straight inlays and inserted in the textile in weft direction. The reinforcement layouts of these specimens were chosen to match the geometric reinforcement content of the directly knitted reinforcement specimen in warp direction (i.e. ca. 0.50%). The reinforcement ratio ($\rho _t$) for the specimens with directly knitted reinforcement and straight inlays can be found in Table 2. In the specimens with inlays, a thin non-structural acrylic yarn was used for the base knitted textile, as this serves only as holder for the inlays. Although it is technically possible to implement inlays in the warp direction (creating a quasi-woven grid within the knitted base textile), this feature was not used and these specimens were only tested in weft direction. The general load-bearing mechanism is the same in both directions for straight rovings since there is no direct interaction between the inlays in warp and weft direction (unlike in directly knitted reinforcement, where the whole textile is made from one continuous yarn) when being loaded in uniaxial tension.

Additionally, spatial features by means of ribs, which are formed by folding the textile and closing the resulting loop (shown in Fig. 2e), were introduced in some configurations of the directly knitted reinforcement and in all configurations with straight inlays to improve the mechanical connection between the reinforcement and the concrete. These ribs were made from aramid yarns with a fineness of 160 tex and were always formed parallel to the weft direction on both sides of the textile. Their approximate average spacing was 35 mm in the directly knitted reinforcement and 40 mm in the textiles with straight inlays. The contribution of the ribs to the reinforcement content was considered in the specimens with directly knitted reinforcement, but neglected in the specimens with straight inlays due to the considerably lower stiffness of the knitted ribs compared to straight rovings.

#### Coating

Two different coating materials were examined. One coating was a highly fluid cement paste consisting of a blended ordinary Portland cement, a polycarboxylate ether based superplasticiser and stabilising nanoparticles at a water-to-binder ratio of 0.24 (mix design courtesy of Dr. Lex Reiter, Chair of Physical Chemistry of Building Materials at ETH Zürich), which was prepared using a mixer providing high shear rates. This type of coating had already been used in the initial applications of KnitCrete [12] and mainly serves for the stiffening of the textile after tensioning when used as flexible formwork. Although the high fluidity of the cement paste leads to a uniform coating thickness over the rough outer surface of the textile, it does not penetrate well into the yarns (i.e. in between the individual filaments), leaving most of the roving core uncoated. Alternatively, a low-viscous two-component epoxy resin [31], as commonly applied for the impregnation of structural textile fibres to improve the mechanical behaviour (e.g. higher strength due to more homogeneous stress distribution within the roving and better robustness against lateral loading [18, 32]), was used. The directly knitted reinforcement was only coated with the cement paste, whereas for the specimens with straight inlays, both cement paste and epoxy coating were examined.

#### Concrete

A fine-grained concrete served as matrix and consisted of sand with a maximum aggregate size of 2 mm (1368 kg/m^3^), Portland cement CEM I (616 kg/m^3^), micro-silica (53.6 kg/m^3^) and superplasticiser (2.91 kg/m^3^) at a water-to-cement-ratio of 0.41, which were mixed in the laboratory.

The flexural tensile strength was determined on small prisms (40 mm $\times$ 40 mm $\times$ 160 mm) according to EN 196-1 [33] in 3-point-bending tests (span of 100 mm); the uniaxial cube compressive strength was obtained from compression tests on the resulting two halves of each prism (loading surface of 40 mm $\times$ 40 mm). The tests on these concrete samples were performed after 8 and 13 days on four different batches (three prisms per batch), where no significant differences were measured. The uniaxial tensile strength was obtained using the conversion formula from fib Model Code 2010 [34], where the flexural tensile strength of 9.5 MPa (standard deviation of 0.7 MPa) resulted in a mean value of 4.2 MPa. The mean compressive strength was 82.7 MPa with a standard deviation of 3.5 MPa.

#### Manufacturing of specimens

The knitted textiles (both directly knitted reinforcement and textiles with straight inlays) were manually tensioned in a wooden frame to reduce deformations of the fabric during application of the coating and, thereby, to minimise any unwanted eccentricities in the specimen. The pre-stressing of the reinforcement does not significantly influence the structural behaviour since the pre-stressing force is very low compared to the tensile strength of the fibres. Both types of coating (cement paste and epoxy) were applied on the tensioned textiles with paint brushes; in the configurations with spatial features, the bond ribs remained uncoated to guarantee direct contact with the concrete. The coated fabrics were cured before casting for three days in a climate chamber ($20\,^{\circ }\hbox {C}$ and 90% relative humidity) in case of the cement paste, and for one day at room temperature in case of the epoxy coating. The specimens were cast in wooden formworks, whose inner faces were sealed with a plastic foil to create a smooth surface texture. The manufacturing followed the principle of the laminating process known from conventional textile reinforced concrete [35]: the coated reinforcement was embedded onto a first bottom layer of wet concrete with a thickness of approximately 5 mm and covered with another layer of 5 mm concrete, which were compacted on a shaking table. The vibration was continued during the placing of the reinforcement (for approximately 30 seconds) to minimise air voids, thereby ensuring a continuous connection between the concrete and the closed surface of the coated textiles. The relatively large variation of the specimen thickness (between 12 and 16 mm) resulted from different thicknesses of the coated textiles (depending on the knitting pattern and coating type) and the higher geometric error margin of the laminating procedure. The specimens were stored in a climate chamber for at least 8 days to harden. The material tests described in Sect. 2.1.3 showed that the strength development of the concrete had been mostly completed, ensuring the comparability of the specimens regarding material properties during the testing period.

### Test setup

**Fig. 3:**
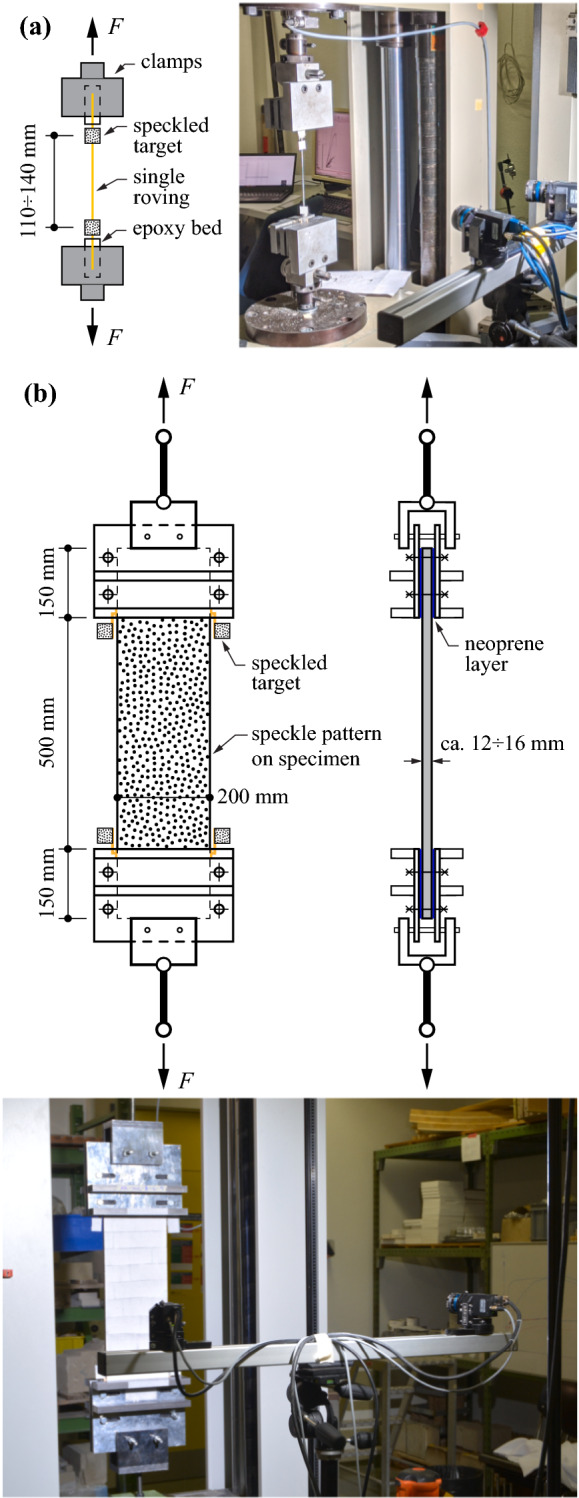
Test setup and specimen dimensions: **a** single rovings and **b** composite tension ties

All experiments were carried out in the structures laboratory at ETH Zürich. The tests aimed at the characterisation of the load-deformation behaviour and the crack kinematics of the weft-knitted textile reinforcement. Besides the concrete tension ties, various series of individual rovings with and without coating were tested. The schematic overview and the actual test setup for both single rovings and composite tension ties are shown in Fig. 3.

#### Single rovings

The material properties of the fibres were obtained from uniaxial tension tests on individual rovings. To study the effect of the impregnation technique, the rovings were coated with epoxy either using a paint brush or being fully immersed in the resin. While the application of the epoxy coating with a brush on the outer surface of the textile corresponds to the actual manufacturing technique (described in Sect. 2.1.4), the immersed rovings represent the reference case where the full impregnation of the roving (i.e. penetration of the epoxy into the roving core) was achieved. Additionally, a series without any coating (which corresponds to the mechanical behaviour of textiles coated with cement paste as described in Sect. 2.1.2) was examined for each fibre type and fineness. Both ends of the rovings were embedded in an epoxy bed to ensure proper anchorage and to minimise the lateral pressure on the fibres introduced by the fixation clamps of the testing machine (Fig. 3a). The free length of the specimens varied between 110 and 140 mm. For each individual configuration, six samples were loaded in a Zwick 10 kN universal testing machine at a controlled displacement rate of 1 mm/min.

#### Textile reinforced concrete tension ties

Following the recommendations of RILEM TC 232-TDT [29], the specimens were clamped with stiffened plates over a length of 150 mm on both sides of the specimen resulting in a free length of 500 mm (Fig. 3b). A 2 mm layer of neoprene was put between the element and the clamping plates to compensate for any irregularities of the concrete surface. The plates were fixed with bolts at a torque of 30 Nm resulting in a notional average clamping pressure of 1.8 MPa. The use of threaded rods and spherical bearings to connect the plates to a Zwick 200 kN universal testing machine resulted in hinged-hinged boundary conditions, which served to minimise eccentricities in the load introduction. The controlled displacement rate was adjusted during the test according to the following scheme:

- Pre-cracking 0.15 mm/min
- During crack formation 0.5 mm/min
- After full crack formation 1.0 mm/min

### Instrumentation

The force was directly measured from the load cells installed in the Zwick universal testing machines. Moreover, for both the single rovings and the reinforced concrete tension ties, 3D-digital image correlation was applied for the assessment of the deformations using a pair of high-resolution cameras (1920$\times$1200 px for single rovings and 4096$\times$3000 px for tension ties) at a stereo angle of approximately $30^{\circ }$. The focal length of the lenses was 25 mm. The correlation was performed with the commercial software ‘VIC-3D’ (Correlated solutions Inc. [36]). In the single roving tests, speckled targets were directly attached to the roving to enable the measurement of the mean elongation. A randomly dark-speckled pattern with a speckle size of 0.7 mm was applied on one face of each concrete specimen to enable the assessment of the quasi-continuous displacement field. Additionally, small plates with a printed speckle pattern were directly attached on the side edges of the specimen just behind the start of the clamped area, as shown in Fig. 3b, to mitigate potential distortions of the measurements by debonding and spalling of the concrete cover close to failure when directly assessing the average strains on the concrete surface. A zero displacement test according to [37] and [38] was performed, resulting in an average noise level of $\sigma (V)$ = 0.0014 mm for the displacement and $\sigma (\varepsilon _{yy}) = 181 \mu \varepsilon$ for the strains in direction of load introduction. These values were achieved with the following correlation parameters: subset size = 15 px, step size = 4 px and strain filter size = 9.

The measurement of the crack kinematics were based on the results of the digital image correlation for quasi-continuous displacement and strain fields over the visible face of the specimen. The open-source software ‘Automatic Crack Detection and Measurement (ACDM)’ [39] developed at ETH Zürich was used for the evaluation of the cracking pattern and the crack widths.

## Experimental results

This section summarises the observations and presents the results of the measurements from the tests on the single rovings, composite elements with directly knitted reinforcement, and composite elements with straight inlays. The analysis of the specimens with directly knitted reinforcement mainly investigated on the general load-deformation behaviour and the failure modes of this new type of reinforcement, exploring the peculiarities arising from different knitting patterns. In the experiments on tension ties with straight inlays, a more detailed comparison of the mechanical behaviour due to different fibre materials and coating types was conducted. A special focus was given to the refined measurements and analysis regarding the cracking behaviour and crack kinematics obtained using the digital image correlation-based measurement system.

### Roving strength and stiffness

**Fig. 4:**
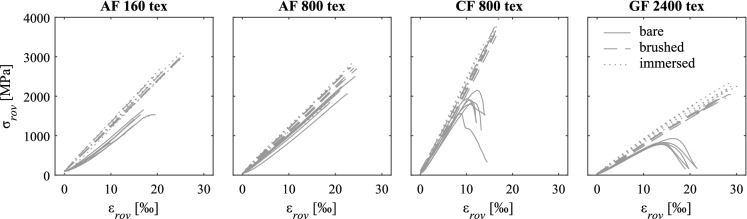
Stress-strain relationship ($\sigma _{rov}$-$\varepsilon _{rov}$) of straight rovings with and without epoxy coating

The strength and stiffness of the individual rovings and the corresponding standard deviation of the measurements were obtained from the stress-strain-relationship depicted in Fig. 4 and are summarised in Table 1. The stresses ($\sigma _{rov}$) were obtained by dividing the measured force of the testing machine by the cross section of the roving ($A_{rov}$). The mean strains ($\varepsilon _{rov}$) were calculated from the total elongation measured directly on the roving with digital image correlation divided by the free length. The coated (brushed and immersed) rovings normally displayed a considerably higher tensile strength than the uncoated (bare) rovings. Only the strength of aramid fibres with a fineness of 800 tex did not seem to be affected by the coating type. The differences between the brushed and immersed rovings were very small and there was no clear tendency regarding strength (particularly, considering the low scatter of the results). The coated aramid rovings with a fineness of 160 tex exhibited a higher tensile strength than the coated thicker rovings (800 tex) of the same material: in bundled strands of multiple filaments, the stresses are transferred via inter-fibre friction, where they continuously decrease from the outer circumference towards the core of the roving [5]. Thinner rovings exhibit smaller differences in the stress distribution over the cross section, leading to higher nominal stresses at failure [40]. The failure mode was brittle for all roving configurations, in which all fibres ruptured at once at the peak load. However, the uncoated carbon and glass fibre rovings displayed a decrease in stiffness before reaching the maximum stresses followed by a softening behaviour with increasing deformation, where the fibres within the roving progressively failed. The tensile strength of all rovings was smaller than the filament strength given by the manufacturer (utilisation of 30–70% for uncoated and 70–97% for coated yarns), which was probably caused by inhomogeneous stress distribution within the roving cross section, and eccentricities in the test setup and the alignment of the fibres within the roving (due to torsion and undulation of the individual filaments), leading to a progressive failure once the first fibre reached the tensile strength. The differences in stiffness caused by the coating type were smaller; even the uncoated rovings reached relatively high Young’s moduli. However, coated rovings displayed higher Young’s moduli than uncoated yarns and there was a slight tendency towards immersed fibres displaying higher stiffness than the brushed ones, which was consistent throughout all roving configurations (increase of 10–20% for brushed rovings and 15–25% for immersed rovings when compared to stiffness of uncoated rovings).

### Tension ties with directly knitted reinforcement

**Fig. 5:**
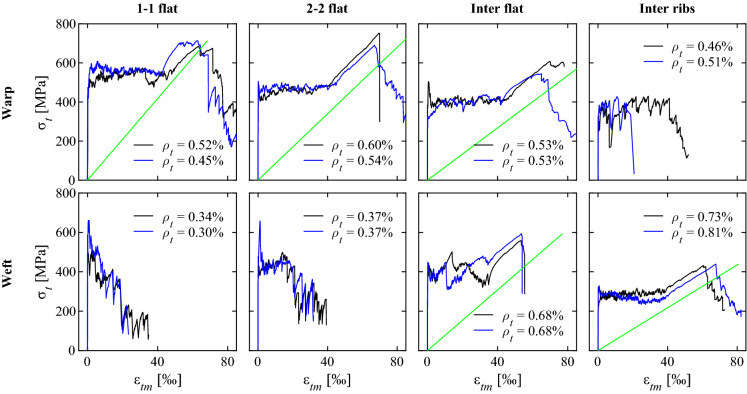
Stress-strain relationship ($\sigma _t-\varepsilon _{tm}$) of specimens with directly knitted reinforcement made from aramid yarns (160 tex) coated with cement paste (textile stiffness obtained from linear regression indicated as green line). (Color figure online)

**Fig. 6:**
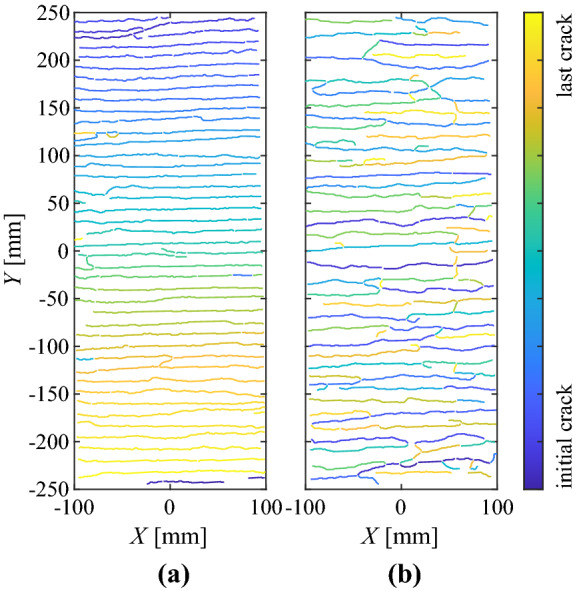
Order of crack development in specimens with **a** directly knitted reinforcement (‘inter flat-2’ loaded in weft direction) and **b** textiles with straight inlays (‘AF-E-2’)

The load-deformation behaviour of the specimens with directly knitted textile reinforcement is shown in Fig. 5. The mean strains ($\varepsilon _{tm}$) were calculated by dividing the total elongation obtained from the speckled targets by the free length of 500 mm. The nominal stresses in the textile reinforcement ($\sigma _t$) were obtained from the total force measured by the load cell, divided by the area of all rovings crossing the section (including the yarns for the bond ribs where applicable). The majority of specimens displayed stable multiple cracking and once crack formation was completed, a strain-hardening phase with a fairly constant slope. The stiffness of the textile was obtained from linear regression in the hardening phase, which resulted in a similar range of Young’s moduli for all knitting patterns ($E_{\text {tc}}$ = 7–10 GPa), shown as green lines in Fig. 5. This stiffness is considerably lower (<10%) than the pure material stiffness due to the interlocked structure of knitted fabrics, which leads to large slippage and rotations between yarns under tensile loading [41]. All specimens with a hardening phase showed a clear effect of tension stiffening: the mean strains of the textile reinforced ties were lower than those of the bare textile at the same load. This is caused by the interaction of the reinforcement with the surrounding concrete (activation of concrete tensile stresses between two cracks). The crack spacing for all specimens was estimated from the crack patterns obtained from ACDM and was between 10 and 20 mm. A summary of all cracking patterns can be found in Fig. 13 in the Appendix. Even in the specimens that displayed hardening behaviour after cracking, the range of the cracked-elastic phase after full crack formation was limited. Due to the fairly low stress and deformation levels reached at failure, a more detailed analysis of the crack kinematics was omitted for the specimens with directly knitted reinforcement. Some specimens exhibited very large crack spacings and premature debonding of the concrete cover (e.g. ‘inter-flat-2’ loaded in warp direction; see Fig. 13 in the “Appendix”) due to large air voids, which had decreased the effective contact surface between coated textile and concrete. These air voids had occurred due to not placing the reinforcement carefully enough while casting (entrapping air between the bottom concrete layer and the coated textile). The nominal cracking stress decreased in some specimens during the multi-cracking phase (e.g. ‘inter-flat-1’ or ‘inter-ribs-2’ loaded in weft direction). Potential reasons for this phenomenon were addressed by Yu et al. [42], who observed this behaviour already in strain hardening cementitious composites. Figure 6a shows the crack development in the specimen ‘inter-flat-2’ loaded in weft direction, where the colour transition corresponds to the order of newly formed cracks. The cracks propagated from one end to the other due to a slight rotation of the specimen caused by eccentricities in the test setup. The resulting out-of-plane moments induced secondary forces on the concrete, reducing its tensile resistance.

**Fig. 7:**
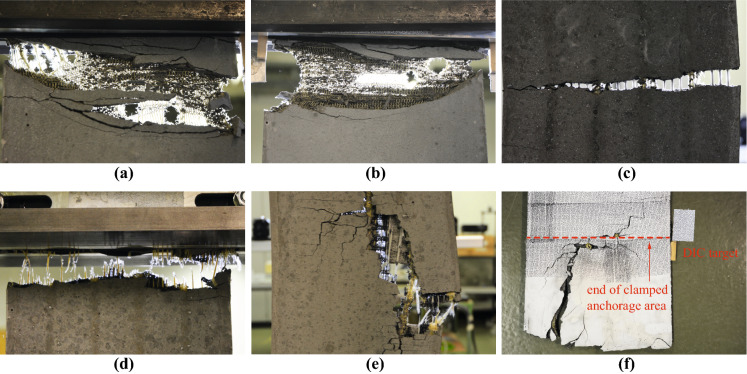
Failure modes of textile-concrete composites: **a** spalling of concrete cover and distortion of directly knitted reinforcement; **b** lateral contraction of single layer textile loaded in weft direction; **c** pull-out failure in specimens with glass fibre inlays and cement paste coating; **d** full and **e** partial rupture of epoxy-coated rovings in specimens with straight inlays; **f** propagation of failure crack into clamped area

The nominal stresses in the textile reinforcement at failure were very low when compared to the tensile strength obtained from the material tests on straight rovings (utilisation lower than 30%). The interlocked structure of the textile introduces highly concentrated lateral forces and sharp bends with low curvature radii in the yarn, which considerably decreases the tensile strength of the fibres [21]. Due to the lacking ductility of the aramid material, there was a brittle failure of the rovings with a steep drop of applied load after reaching the peak stress. However, some specimens displayed a pseudo-softening behaviour (e.g. ‘2-2-flat-2’ or ‘inter-flat-2’ loaded in warp direction), which did not arise from the material properties per se but from progressive rupture of knitted loops in the interlocked textile. The resulting large deformation and distortion of the textile (caused by the local damages and the lateral contraction of the fabric) led to debonding and eventually spalling of the concrete cover (Fig. 7a).

A different behaviour was observed in the single layer textiles (‘1–1’ and ‘2–2’) that were loaded in the weft direction. These specimens failed directly after reaching the tensile strength of the concrete, and deformations concentrated in a single crack. In weft direction, the reinforcement content was considerably lower than in warp direction. However, the utilisation of the fibres was even lower than in the specimens that displayed hardening behaviour. From visual inspection, it seemed that the textile showed a higher degree of lateral contraction when loaded in weft direction, which might have led to premature spalling of the concrete cover and therefore, significant loss of stiffness of the embedded textile.

The specimens with bond ribs failed prematurely shortly after initial cracking when loaded in warp direction. The ribs ran in parallel to the weft direction, which introduced a local weakness in the textile since the integration of bond ribs requires a denser knitting pattern with smaller loop size and tighter knots. This effect did not occur in weft direction — where the ribs were oriented in loading direction — as the specimens displayed a stable hardening behaviour until a brittle failure occurred. In this case, the bond ribs seemed to improve the behaviour of the textile-concrete-composite as they reduced the tendency of spalling of the concrete cover, which normally occurred close to failure. This can be attributed to the mechanical connection created between concrete and reinforcement, which is not present in the specimens without bond ribs, where the interface conditions mostly rely on chemical adhesion in the cold joint. However, the stiffness of the reinforcement with bond ribs was considerably lower when compared to the flat textiles (around 30% lower), which suggests that the ribs are more flexible than the base textile and, therefore, carry less load. The stress-strain-relationship suggests that the flat specimens exhibited a more pronounced tension stiffening effect compared to the specimens with bond ribs, which, since the crack spacing was similar for both configurations, would imply that the bond shear stress was in fact higher without ribs. However, more investigations are needed to confirm this observation since the hardening phase was not very pronounced and specimens failed still rather shortly after full crack formation.

### Tension ties with straight inlays

**Fig. 8:**
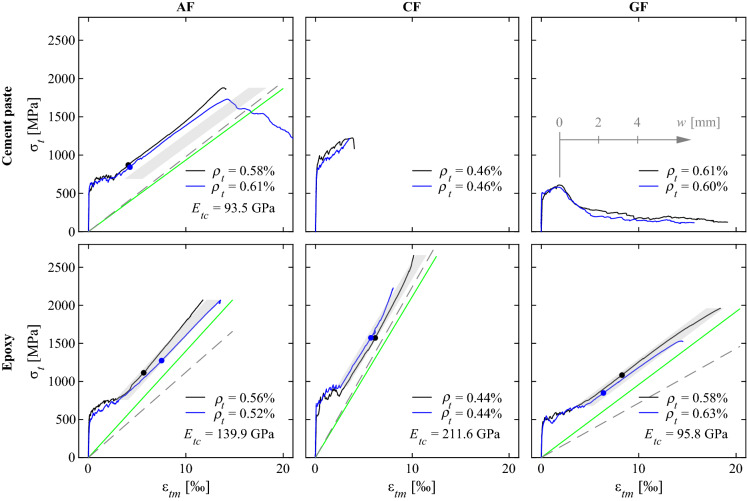
Stress-strain relationship ($\sigma _t-\varepsilon _{tm}$) of specimens with straight inlays and bond ribs (green line: textile stiffness $E_{tc}$; dashed line: roving stiffness $E_{rov}$; grey area: post-diction using Tension Chord Model; filled circles: crack stabilisation according to crack spacing development). (Color figure online)

Figure 8 shows the load-deformation behaviour for the specimens with straight inlays and bond ribs. For the calculation of the textile stresses ($\sigma _t$), only the cross sectional area of the straight inlays were considered since (1) the base textile was made from a non-structural yarn with negligible strength and stiffness and (2) the knitted ribs had a much lower stiffness compared to straight rovings. The bond ribs were still introduced due to their beneficial effect regarding spalling of the concrete cover, which had been observed in the specimens with directly knitted reinforcement (Sect. 3.2 and Fig. 7a).

#### General load-deformation behaviour and failure modes

Most specimens displayed a strain-hardening behaviour after cracking with multiple uniformly distributed cracks. The utilisations of the fibres were much higher than in the specimens with directly knitted reinforcement since the fibres are not bent. However, the textile stresses at failure still did not reach the full tensile strength obtained from the single roving tests. This lower utilisation in textile reinforced concrete arises from several reasons, which have been documented in existing literature for woven fabrics and are also applicable to the reinforcement with straight inlays in the present study: (1) Stresses are transferred from the concrete to the roving over its outer circumference and distribute within the cross section of the yarn via internal friction between individual filaments. Depending on the type of coating and the depth of penetration into the roving, the stress profile over the cross section of the yarn can range from almost uniform (in case of fully impregnated roving) to highly non-homogeneous, where the core fibres might not be loaded yet while the outer fibres of the roving already reach the tensile strength [4, 40]. (2) Even with hinged-hinged boundary conditions in the test setup, it is not possible to guarantee a perfect alignment of the specimen and still some eccentricities might occur. Once a crack forms, the rovings are deviated at the crack edges, which introduces highly concentrated lateral forces on the reinforcement, eventually damaging the yarn and causing a reduction of tensile strength [15]. (3) The filaments might be damaged during several steps in the production sequence (manufacturing of the textile, storage, transportation, installation on site, casting) [43].

In all these cases, the brittle nature of the fibre material prevents a redistribution of stresses after the first fibre reaches its tensile strength. Consequently, the fibres will progressively fail, making the tension ties prone to premature failure due to local material deficiencies. Evidently, the choice of proper coating and, thus, impregnation of the rovings is paramount to improve the performance and robustness of the textile reinforcement. The experimental results of this study showed that epoxy coating (E) had a more beneficial effect on the structural behaviour. With cement paste coating (C), the specimens with carbon and glass fibre rovings failed shortly after initial cracking and only a few cracks had formed (premature failure of CF-C and GF-C during multi-cracking phase as shown in Fig. 8). The glass fibre rovings showed a distinct softening behaviour with deformations concentrating in a single crack (grey axis in Fig. 8; failure crack in Fig. 7c). From visual inspection of the rovings after testing, it could be seen that only the outer fibres of the yarn were ruptured, and the core had been pulled out of the sleeve of fibres. This type of failure is also known in conventional textile reinforcement and mainly occurs due to insufficient stress transfer among fibres within a roving [18] and higher sensitivity of carbon and glass fibres to lateral loading, making it prone to damage from local lateral pressure at the crack edges [4]. When coated with cement paste, only the aramid rovings displayed strain hardening after full crack formation, where one of the specimens exhibited a distinct softening behaviour after reaching the peak load. In contrast, all fibres were typically either fully (Fig. 7d) or partially (Fig. 7e) ruptured in the epoxy-coated textiles, exhibiting a brittle failure without any post-peak residual stresses. The utilisations of fibres in the straight inlays with epoxy coating were between 50% and 70% of the filament strength (indicated by fibre manufacturer), which is in a similar range as for conventional textile reinforcement (as will be discussed in Sect. 5), and between 60% and 100% of the tensile strength obtained from the single rovings with brushed epoxy coating.

#### Textile stiffness

The textile stiffness ($E_{tc}$) of the reinforcement that exhibited a hardening behaviour was obtained from linear regression of the stress-strain relationship in the range of serviceability limit state (between 1/2 and 2/3 of textile stress at ultimate load $\sigma _{tu}$), as summarised in Table 3. They were compared to the roving stiffness obtained from the single roving tests, where for the cement paste-coated specimens, the bare stiffness and for the epoxy-coated specimens, the stiffness of the brushed rovings was taken. The average of the two twins per configuration is denoted as solid green line and the roving stiffness as dashed grey line in Fig. 8. Whereas the specimens with aramid fibres and cement paste coating, and the specimens with carbon fibres and epoxy coating exhibited slightly lower stiffness than the single rovings (0.91–0.97 of $E_{rov}$), aramid and glass fibres rovings with epoxy coating displayed considerably higher Young’s moduli, even exceeding the roving stiffness. In case of the epoxy-coated aramid inlays, the twins displayed a large difference in stiffness, where one specimen correlated quite well with the roving stiffness (1.13$\cdot E_{rov}$), but the other exhibited a much more pronounced hardening (1.38$\cdot E_{rov}$). In this specimen, the bond ribs most likely had contributed to the load-bearing behaviour. Due to the low-viscous epoxy and its manual application, it was difficult to ensure that only the areas in the textile with inlays were coated. From the observations on the directly knitted reinforcement, it appeared that the bond ribs had an even lower stiffness than the knitted textile ($E_{tc} = 7-10\ \hbox {GPa}$). Impregnating the aramid yarns in the ribs and therefore, stiffening their knitted structure — for which already a little amount of coating would be sufficient — could have caused the significant increase of overall stiffness of the tension tie. In some specimens (‘AF-C-1’ and ‘CF-E-1’), there seemed to be an increase of stiffness close to failure. The visual inspection of the broken specimen after testing revealed that the failure crack had propagated into the clamped area (Fig. 7f), where it was not possible to capture the deformations with the DIC measurements. Therefore, the deformation measurements close to failure of these specimens have to be treated with caution. The specimens with glass fibre inlays and epoxy coating both exhibited a higher stiffness than the single rovings (+38% and +29%). However, the stress-strain-relationships in Fig. 8 show that there was a decrease in stiffness of the textile towards the failure load. This effect might indicate that cracking had not yet been stabilised, which will be addressed in more detail in the crack formation analysis. When applying the linear regression in the latter stage of the hardening phase (between 0.8 and 1.0 of failure load), the measured stiffnesses of the textile composites with glass fibre inlays were only marginally higher (+9% and +16%) than the single roving stiffness, which lies in a more plausible range.

**Table 3:**
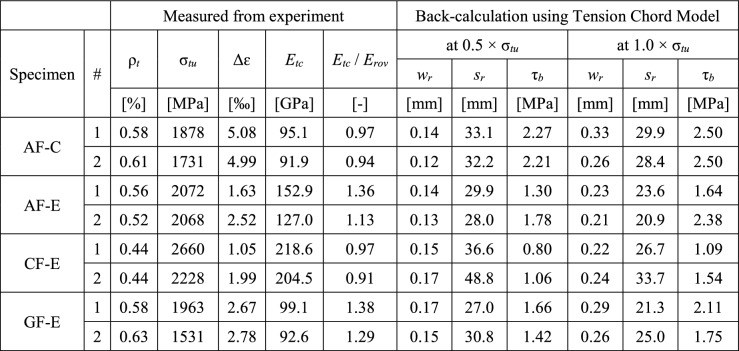
Crack kinematics and back-calculated bond stresses for specimens with straight inlays

#### Crack kinematics

**Fig. 9:**
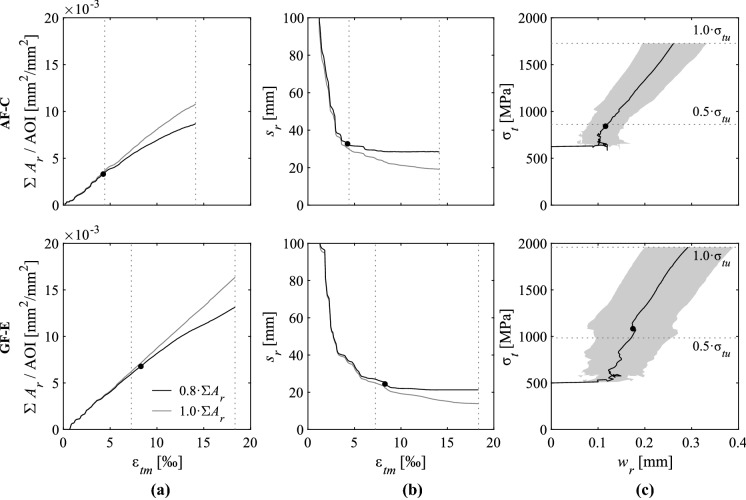
Evolution of crack kinematics: **a** Total crack area $A_r$, **b** crack spacing $s_r$ and **c** mean and 10-90% quantiles (grey area) of the crack width $w_r$ for specimens with aramid inlays coated with cement paste (‘AF-C-2’) and with glass fibres inlays coated with epoxy (‘GF-E-1’), respectively

The cracking pattern and kinematics were determined with ACDM based on the quasi-continuous displacement and strain field measurements of DIC. The results of two selected specimens (aramid inlays with cement paste coating, ‘AF-C’, and glass fibre inlays with epoxy coating, ‘GF-E’) are shown in Fig. 9. The summary of all specimens with strain-hardening behaviour can be found in Fig. 14 in the Appendix. To assess the major cracks that mainly contribute to the overall deformations, the total crack area ($A_r$) divided by the area analysed in the digital image correlation (‘area of interest’) was plotted against the mean strains [44] (see Fig. 9a). In this study, the crack area was defined as the component of the crack opening in the loading direction multiplied by the horizontal projection (perpendicular to the loading direction) of the crack length. The measurement was taken at every data point along the crack and summed up for the calculation of the total crack area. The linear relationship between mean strains and total crack area proved that the structural behaviour was governed by the cracks and that concrete deformations were negligible. Furthermore, it served as plausibility check for the fully automated crack detection and processing procedure described in this section (see Fig. 9 and Fig. 14 in the “Appendix”). The number of cracks crossing the full section was determined from the total crack length divided by the width of the area of interest (AOI). The average crack spacing ($s_r$) was consequently obtained by dividing the free length of the specimen by the number of cracks (Fig. 9b). Only the measuring points that contributed to 80% of the total crack area at failure and having openings larger than 0.05 mm were considered to compute the statistical values of the crack widths shown in Fig. 9c, Table 3 and Fig. 14 in the “Appendix”. These are denoted as ‘major cracks’; this procedure served to filter out fine cracks that only formed near failure, which otherwise would have distorted the measurements. Comparing the measurements with all cracks (grey lines) and only the major cracks (black lines), the curves for both total crack area and crack spacing start diverging at approximately 50% (Fig. 9a and b). The crack spacing only decreases marginally until failure when considering 80% of the total crack area. In this study, the point of crack stabilisation was determined once the crack spacing was within 15% of the final crack spacing at failure, which is denoted as filled circles in the load-deformation curve in Fig. 8 and in the crack kinematics in Fig. 9. The mean crack width ($w_r$), as well as the 10% and 90% quantiles, considering only the major cracks are plotted in Fig. 9c. During the initial crack formation phase, the crack width did not significantly increase , while in the hardening phase, the crack width almost linearly increased with the textile stresses.

**Fig. 10:**
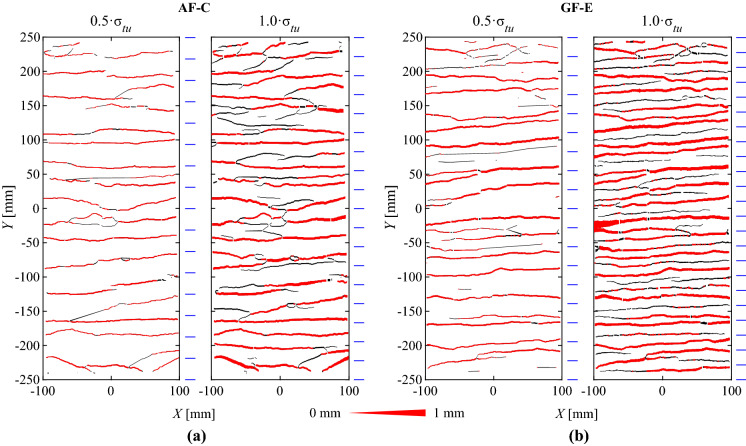
Cracking patterns (with line thickness corresponding to crack opening; major cracks contributing to 80% of total crack area in red) and average crack spacing (indicated by blue lines) at 50% of the failure load and close to failure for specimen with **a** aramid inlays coated with cement paste (‘AF-C’) and **b** glass fibre inlays coated with epoxy (‘GF-E’). (Color figure online)

Figure 10 shows the cracking pattern and the corresponding kinematics (where the line thickness represents the crack opening) at 50% and at 100% of the failure load for the specimens that were examined in Fig. 9; major cracks that were considered for the statistical analysis of the crack kinematics are marked in red. In the specimens with aramid inlays and cement paste coating (‘AF-C’), most of the newly formed cracks until failure were secondary branching cracks (black lines) and therefore did not significantly contribute to the total elongation of the specimen (Fig. 10a). In case of glass fibre inlays with epoxy coating (‘GF-E’), the crack spacing had not stabilised at 50% of the ultimate load and therefore, further major cracks formed until failure (Fig. 10b). The summary of the cracking patterns close to failure for all specimens with stable multiple cracks can be found in Fig. 15 in the “Appendix”. The average crack spacing and corresponding crack widths at 50% and 100% of the failure load for all specimens are summarised in Table 3. The cracks widths are mostly dependent on the crack spacing and on the stiffness of reinforcement. The resulting measurements were in a similar range for all specimens at the same relative load level (normalised to the peak load) due to the different magnitude of loads that the specimens reached at failure.

The experiments showed that new major cracks still form in the linear branch of the stress-strain-relationship after the multi-cracking phase, which was mostly observed in the epoxy-coated specimens (see Fig. 14 in “Appendix”). In these specimens, the crack spacing stabilised at higher loads (up to 70% of ultimate load in case of carbon fibres inlays with epoxy coating), which is also clearly visible in development of crack width since the slope of the stress-crack width relationship was constant only after this load level.

In the specimens with straight inlays (Fig. 6b), cracks formed more randomly along the specimens compared to the directly knitted reinforcement, where — as mentioned in Sect. 3.2 — the cracks propagated from one end to the other (Fig. 6a). Since the manufacturing procedure and the test setup were the same for both reinforcement types, the general imperfections should essentially be similar. The difference in cracking behaviour may, however, be explained by larger rotations in the specimens with directly knitted reinforcement due to their lower stiffness. This would lead to a more distinct straightening effect due to potential eccentricities and, therefore, to higher secondary forces, causing cracks to start forming near one end.

## Tension Chord model for textile reinforced concrete

**Fig. 11:**
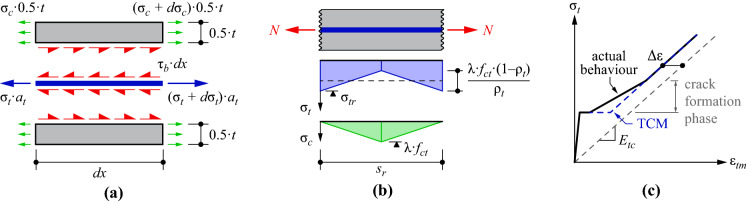
Tension Chord Model: **a** Equilibrium on differential element; **b** crack element with corresponding stresses in reinforcement and concrete; **c** resulting tension shift in stress-strain-relationship

All specimens with strain hardening behaviour displayed a clear effect of tension stiffening, which is caused by the interaction of the reinforcement with the surrounding concrete. Part of the stresses in the reinforcement, which carries the full tension at the cracks, are transferred to the concrete between the cracks via bond shear stresses along the interface of the two materials. This causes the average deformations of the composite ($\varepsilon _{tm}$) to be smaller than the deformations of the bare textile ($\varepsilon _t$), as seen in the stress-strain-relationships (Fig. 8). The bond shear stresses are typically defined as bond stress-slip-relationships since relative deformations between the reinforcement and the concrete (i.e. slip $\delta$) are needed to activate bond stresses. Generally, this requires a numerical solution of the differential equation that is obtained from equilibrium on the composite elements, as shown in Fig. 11a, i.e.:

1 $$\begin{aligned} \frac{\partial ^2\delta }{\partial x^2}=\frac{2}{a_t}\cdot \left( \frac{1}{E_{tc}}-\frac{\rho _t}{E_c}\right) \cdot \tau _b(x) \end{aligned}$$

where $\tau _b$ is the bond shear stress; $\rho _t$ is the geometrical reinforcement content̄; $a_t$ is the cross sectional area of the reinforcement; $E_{tc}$ and $E_c$ are the stiffnesses of the textile reinforcement and the concrete, respectively. For the formulation of the free body diagrams in Fig. 11a, it was assumed that, despite the ribs, the interface area between textile and concrete was completely flat. The Tension Chord Model [28] proposed a simplification of the bond-slip-relationship for steel reinforcement, assuming the bond shear stresses to be constant as long as the reinforcement is elastic. This allows solving the problem solely based on equilibrium, leading to linear distributions of the stresses in the reinforcement and in the concrete (Fig. 11b). This results in a constant reduction of strains in the stress-strain-relationship when compared to the bare textile, as illustrated in Fig. 11c. Although this is a great simplification of the highly complex and non-linear bond mechanism, extensive research in literature for concrete structures with steel reinforcing bars (e.g. [45, 46]) has shown that this assumption leads to very reliable results for the global structural behaviour (i.e. load-deformation behaviour). The tension shift ($\Delta \varepsilon$) can be formulated as follows, where $\lambda$ is the ratio of the actual crack spacing to the maximum crack spacing and lies between 0.5 and 1.

2 $$\begin{aligned} \Delta \varepsilon =\frac{\lambda \cdot f_{ct}\cdot \left( 1-\rho _t\right) }{2\cdot \rho _t\cdot E_{tc}} \end{aligned}$$

### Post-diction of the experimental results

To validate whether the Tension Chord Model reasonably represents the behaviour of concrete elements with weft-knitted textile reinforcement, a post-diction of the experimental results using Eq. (2) was conducted. In this analysis, the stiffness of the textile ($E_{tc}$) was taken from the experiments (mean value of two specimens per configuration). The reinforcement ratio $\rho _t$ was defined as the area of the straight rovings divided by the gross concrete cross section. The tensile strength $f_{ct}$ was taken from the material tests on the small prisms described in Sect. 2.1.3. The results are shown in Fig. 8 as grey area, which represents the theoretically feasible range of $\lambda$. The epoxy-coated specimens all show a good correlation with the model. However, the tension shift for the cement paste-coated aramid reinforcement is underestimated. The deviation might be partially caused by an underestimation of the tensile strength of the concrete since the conversion factor from flexural to tensile strength taken from fib Model Code 2010 [34] is rather low when compared to the actual cracking load achieved in the composite tension chords. However, this would also affect the specimens with epoxy coating, which might worsen their post-diction. Therefore, it seems that the assumption of a constant bond shear stress at the interface is less adequate to the cement paste-coated reinforcement. The visual inspections of the yarns after failure showed that the cement paste did not penetrate well into the rovings and only coated the outside surface of the yarn whereas the epoxy achieved a better penetration depth. The cement paste therefore creates another interface between the coating and the loose fibre core, which would require a more refined bond stress-slip relationship.

### Estimation of bond shear stresses at the interface between textile and concrete

Besides the post-diction of the load-deformation behaviour of the experiments, the Tension Chord Model can be used to estimate the average nominal bond stress with the actual tension shift and crack spacing obtained from the experiments:

3 $$\begin{aligned} \tau _b=\frac{2\cdot a_t\cdot E_{tc}\cdot \Delta \varepsilon }{s_r} \end{aligned}$$

Equation (3) is formulated for the specific geometry of the weft-knitted textile reinforcement, whose interface with the concrete is characterised by a flat surface on both sides of the textile (while the bond shear stresses are transferred over the circumference of the steel reinforcing bars or the rovings in conventional textile reinforcement). The textile stiffness ($E_{tc}$) and the tension shift ($\Delta \varepsilon$) were derived by linear regression in the range of serviceability loads (1/2$\ldots$2/3 of ultimate load). The crack spacing ($s_r$) was taken at 50% and 100% of the ultimate load considering only the contribution of the main cracks (Fig. 14 in Appendix). The results are summarised in Table 3. The cement paste coating displayed higher bond stresses than all specimens with epoxy-coating. There seems to be a slight trend for higher bond stresses with lower stiffness of the reinforcement; although the differences between aramid and glass fibre are very small, carbon fibre inlays have the lowest value. There is a small increase of bond stresses at higher load. Since the calculation of bond stresses is not only dependent on the crack spacing but also the textile stiffness and tension shift, the Tension Chord Model is only applicable for determining $\tau _b$ once the cracking is fully stabilised. The bond stresses obtained at failure load are between 1.1 MPa and 2.5 MPa ($26\%-62\%$ of concrete tensile strength $f_{ct}$), which is much lower than in conventional steel reinforcing bars, where usually a value of 2$\cdot f_{ct}$ leads to a reasonable estimation of the mean strains. Whereas in conventional deformed reinforcing bars, the ribs ensure an effective stress transfer (via compression struts) [45], it mainly depends on adhesion and friction in textile reinforcement.

## Comparison with conventional textile reinforcement

**Fig. 12:**
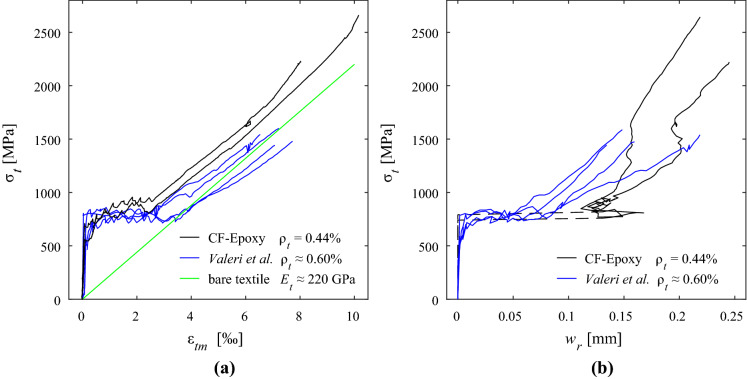
Comparison with conventional textile reinforcement (data from Valeri et al. [47]): **a** Stress-strain relationship of textile reinforced composites; **b** average crack width development for increasing load

**Fig. 13:**
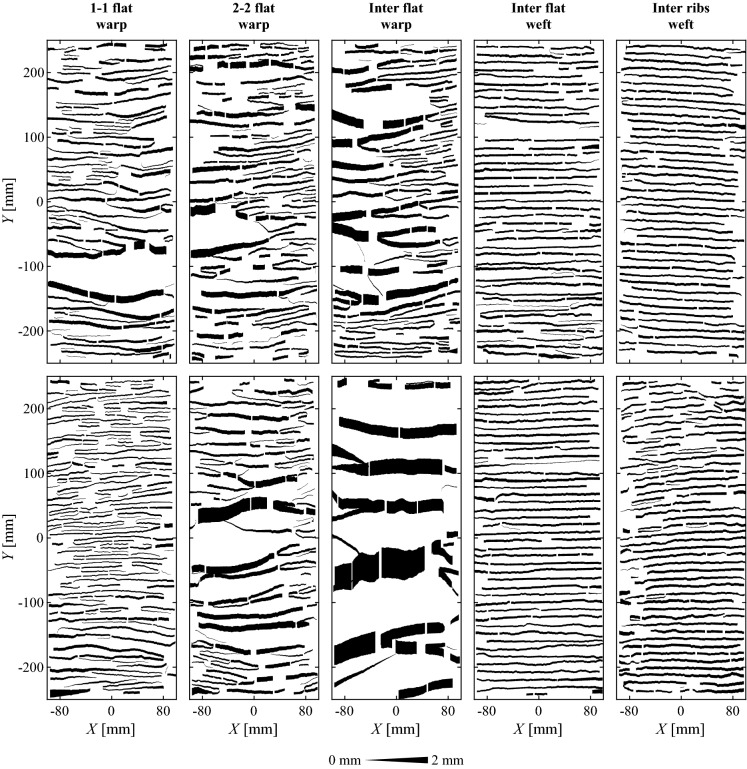
Cracking pattern close to failure (with line thickness corresponding to crack opening) for all specimens with directly knitted reinforcement from aramid yarn exhibiting strain-hardening behaviour (unreliable measuring points according to ACDM are omitted)

**Fig. 14:**
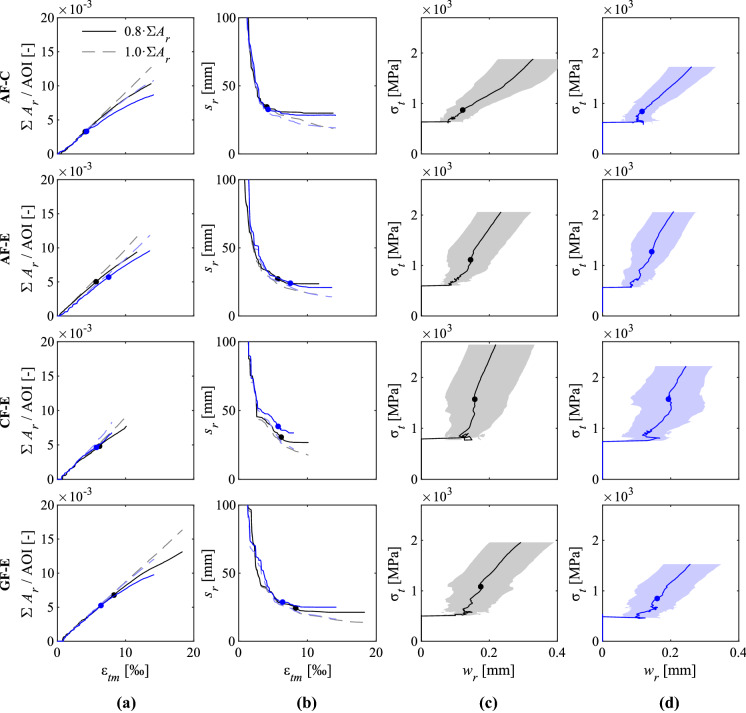
Evolution of crack kinematics: **a** Total crack area $A_r$, **b** crack spacing $s_r$ and **c**/**d** crack width $w_r$ for all specimens with straight inlays and bond ribs that exhibited hardening post-cracking behaviour

**Fig. 15:**
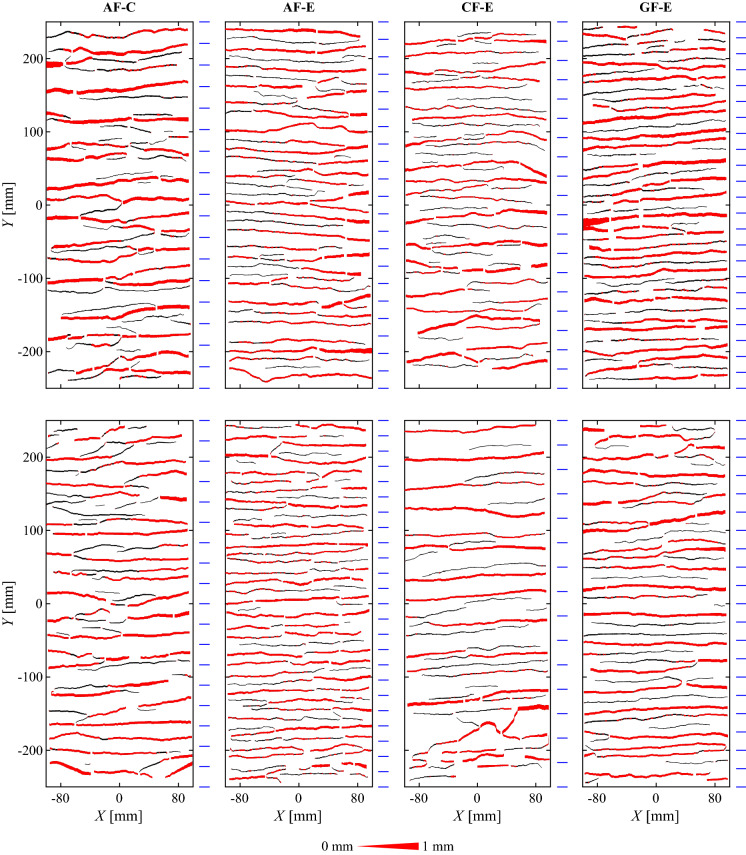
Cracking patterns close to failure (with line thickness corresponding to crack opening; major cracks contributing to 80% of total crack area in red) and average crack spacing (indicated by blue lines) for all specimens with straight inlays exhibiting strain-hardening behaviour. (Color figure online)

Figure 12 compares the load-deformation behaviour of the specimens with straight inlays made from carbon fibres and epoxy coating with the results from Valeri et al. [47]. That study examined textile reinforced concrete ties with similar geometry (thickness of 16 mm, free length of 500 mm, but a width of only 100 mm) in uniaxial tension and investigated different reinforcement layouts, mainly varying the reinforcement content but also the coating type. The specimens chosen for comparison had a similar amount of reinforcement ($\rho _t\approx 0.60\%$), which consisted of two layers of woven grids made from carbon fibre. The rovings had an additional quartz-sand coating to improve the bond conditions. Pull-out tests on single rovings yielded a peak bond shear stress of 3 MPa. The concrete had a maximum aggregate size of 1.6 mm, and exhibited a tensile strength of 4.1 MPa and a cylinder compressive strength of 107.8 MPa after 28 days.

The weft-knitted textile with straight inlays reached considerably higher stresses at ultimate load (+60% in average) than the woven textiles. Besides the different type and application of coating, which is difficult to compare directly, the undulation of the rovings in woven textiles further reduces the strength of the fibres due to the induced lateral pressure on the roving. Moreover, Valeri et al. used clamped boundary conditions to fix the specimens to the testing machines, which might have caused some eccentricities and additional deviation at the crack edges. Existing literature (e.g. [43, 48, 49]) indicates that — depending on the fibre material, coating type and reinforcement layout — the nominal stresses in the textile normally reach between 40% and 70% of the filament strength, where besides rupture of the fibres, the formation of longitudinal cracks along the rovings and premature debonding is a frequently occurring failure mode. In other studies (e.g. [15]), the more locally concentrated force transfer in conventional textile reinforcement caused high splitting stresses, which often led to premature failure due to the formation of longitudinal cracks along the roving. This type of failure did not occur in the present study with knitted textiles. However, longitudinal cracks formed along the bond ribs after reaching the ultimate load and the partial rupture of the inlays in the specimens with epoxy-coated glass fibre inlays (‘GF-E’) as shown in Fig. 7e.

The stress-strain relationship is very similar for weft-knitted and conventional textile reinforcement (Fig. 12a). Both exhibited a stable hardening phase after crack formation, and the stiffness corresponded well with the material tests. However, the mean strains (at the same load levels) of the specimens with weft-knitted textiles were generally lower than those of the specimens with conventional textile reinforcement. This effect of tension stiffening is significantly influenced by the bond conditions and the interface geometry between reinforcement and concrete.

The crack spacing of thin concrete elements with conventional textile reinforcement is mostly governed by the grid spacing since the lateral rovings introduce a weakening of the concrete cross section. The measured crack spacing in the conventional reinforcement used by Valeri et al. (grid of 20 mm $\times$ 20 mm) was between 20 and 30 mm, and thus, slightly smaller than in the specimen with weft-knitted textiles (26 and 33 mm). The average crack width of the specimens with weft-knitted textiles was larger especially at lower load levels (Fig. 12b), which was also due to the fact that crack formation had not stabilised. At higher loads, the development of the average crack width closely resembles a linear continuation of the conventional textile reinforcement, which indicates a good correlation in general load-deformation behaviour of these two reinforcement types. Many studies in literature (e.g. [18, 24–26, 50]) have attempted to characterise the bond shear stresses for conventional textile reinforcement, which is — just like the strength and stiffness — highly dependent on the specific reinforcement product (primarily the surface texture of the rovings) where additional features such as sand coating to enhance the mechanical bond conditions have significant influence. The bond stresses for conventional textile reinforcement are typically obtained from pull-out tests, and maximum values amount to $2-5$ MPa. The bond stress-slip relationship usually exhibits a steep increase, reaching the peak stress already at small slip (between 0.01 mm and 0.05 mm), and subsequently decreasing to a residual bond stress (typically around 1 MPa at a slip of approximately $0.5-1.0\ \hbox {mm}$). The bond shear stresses derived from back-calculation of the tension chords seem to be reasonable considering the fact that a constant bond stress-slip relationship was assumed. Although the bond shear stresses of conventional textile reinforcement and the knitted textiles with straight inlays are in a similar range, the contact areas of the two reinforcement types are very different. Conventional textile reinforcement only transfers stresses around the circumference of the roving, but the knitted textiles exhibit a closed surface along the whole width of the specimen. Comparing the conventional textile reinforcement examined in Fig. 12, the contact area of the knitted textile is a magnitude larger (400 mm^2^/mm vs. 40 mm^2^/mm), which eventually leads to much higher force transfer between reinforcement and concrete. Although there is only little literature specifically addressing the effect of tension stiffening in conventional textile reinforcement, the comparison with similar tests in uniaxial tension (e.g. [47], Fig. 12) shows that conventional textile reinforcement when compared to knitted textiles seems to exhibit a much less pronounced decrease of mean strains from the bare textile. Although this can be partially explained by the lower reinforcement content of the specimens with knitted textiles (see Eq. (2)), some of the specimens with conventional textile reinforcement seemed to exhibit hardly any tension stiffening effect. The reinforcement grid of the orthogonal rovings highly influences the crack spacing in these specimens, which can lead to much denser cracking patterns than what would follow from the tensile strength of the concrete according to the Tension Chord Model. This leads to a reduced stress transfer between concrete and reinforcement and, eventually, to a smaller tension shift (see Eq. (3)).

## Conclusions

The objective of this study was to investigate the post-cracking behaviour of concrete tension ties with weft-knitted textile reinforcement. To this end, an experimental campaign consisting of uniaxial tension tests on single rovings and composite elements with directly knitted reinforcement and straight inlays was conducted. The investigations focused on various knitting patterns, various fibre materials, types of coating and spatial features by means of ribs within the textile to enhance the bond conditions at the interface between reinforcement and concrete. Using digital image correlation-based measurement systems and the Automated Crack Detection and Measurement tool, it was possible to assess the cracking patterns and the quasi-continuous measurement of crack kinematics. The Tension Chord Model, which was originally derived for conventional steel reinforcing bars, was adapted to the specific geometry of the flat tension ties, which allows the back-calculation of bond stresses assuming a constant value for the bond stress-slip relationship.

The experimental results show that the post-cracking behaviour of the textile reinforcement highly depends on the knitting pattern. For directly knitted reinforcement, the interlocked structure of the textile creates many sharp bends and, thus, concentrated lateral loads on the yarn. This significantly decreases the tensile strength of the fibrous material, leading to a utilisation below 30% when compared to the filament strength. The stretchiness of the fabric not only reduces the stiffness by one magnitude compared to the material stiffness, but also introduces lateral contraction when loaded in uniaxial tension, which caused spalling of the concrete cover and a premature failure in certain configurations.

Specimens with straight inlays exhibited a much higher utilisation of the yarn in both strength and stiffness. Epoxy coating showed a more beneficial effect compared to cement paste due to its deeper penetration into the yarn, which increased the robustness of the rovings. Carbon and glass fibres with cement paste coating failed shortly after initial cracking, while all epoxy-coated specimens and only aramid inlays with cement paste coating displayed a distinct hardening behaviour, reaching utilisations of around 60-100% compared to the tensile strength obtained from the single roving tests. The high utilisation in terms of stiffness (over 90%) indicates a fairly homogeneous stress distribution within the rovings and supports the hypothesis that the brittle nature of the fibres and concentrated deviation forces at the crack edges are the governing influences for the reduced utilisation of the roving strength. However, the clamping pressure at the supports might have improved the anchorage of the rovings. There might be a reduction of strength and stiffness in loading cases where the reinforcement is solely activated by bond stresses along the outer surface of the roving (e.g. bending beams without end anchorage).

The effect of the integration of ribs into the textile was ambiguous. Although they did not directly improve the bond, they seemed to improve the behaviour regarding spalling of the concrete cover. The contribution to the load-bearing behaviour in tension is not clear yet either. In the directly knitted reinforcement with cement paste coating, their influence seemed to be negligible. Although this also seemed to be the case for most of the configurations with straight inlays, some epoxy-coated specimens exhibited even higher stiffness than the single rovings, which could be attributed to either a delayed stabilisation of the crack formation or the accidental coating (due to the use of a brush) and, therefore, stiffening of the bond ribs made from aramid fibres.

The specimens with hardening behaviour showed stable multiple cracking, which resulted in regular cracking patterns with closely spaced cracks across the section. However, the refined measurement of the experiments showed that new major cracks could still form in the linear branch of the stress-strain-relationship, which was mostly observed in the epoxy-coated specimens. Despite the large total deformations of the specimens, the crack widths stayed well below the common limits under service loads (below $0.2{\text {--}}0.5\ \mathrm{mm}$, depending on code and exposure conditions).

The Tension Chord Model allowed a good representation of the load-deformation behaviour of the specimens with straight inlays and epoxy coating, with only few material parameters having to be known a-priori. The model underestimated the tension shift for the specimens with cement paste coating showing that the assumption of a constant bond stress independent of the slip might not be adequate in this case. The back-calculated bond stress was lower for epoxy coating and seemed to decrease with higher stiffness of the reinforcement, but no clear tendency regarding the slip could be detected. Therefore, further investigations on the bond mechanism are needed, including more refined experiments specifically targeting the capture of the local bond behaviour such as pull-out tests.

Based on this study, the use of directly weft-knitted textiles as reinforcement is not recommended due to the massive reduction in strength of the fibres and large deformations even at serviceability limit state. However, the integration of straight inlays into a base textile made from a non-structural yarn offers great potential as main reinforcement. The general load-deformation behaviour of the weft-knitted textiles with straight inlays is similar to conventional textile reinforcement. Although the bond stresses are lower than with steel reinforcing bars, the larger interface area (due to the closed surface of the coated textile) still leads to a considerable stress transfer between textile reinforcement and concrete, leading to a more pronounced effect of tension stiffening than in conventional textile reinforcement (where the bond shear stresses are transferred over the circumference of the rovings) and thus, a more favourable behaviour in serviceability limit state. However, more investigations are needed regarding the activation of rovings for other loading cases such as bending.

This study focused on the uniaxial tensile response of concrete elements with weft-knitted textile reinforcement. The fundamental findings on this newly developed reinforcement type introduce the foundation for further investigations on more loading conditions such as biaxial membrane forces, bending moments or in-plane shear. The possibility of integrating inlays in both warp and weft directions offers immense potential for use in concrete structures with complex geometry (such as doubly curved shells or folded plates). The greater geometric freedom of the knitting procedure allows a flexible placement of rovings within the base textile (e.g. following the directions of principal forces), which may lead to a more efficient design compared to orthogonally woven arrangements. However, challenges already known from conventional textile reinforced concrete — such as reduced strength due to skewed loading or crack sliding [15, 18], or web splitting and crushing in thin folded elements [51] — may arise, which need to be investigated in further research.

## Data Availability

The experimental data presented in this article was submitted to a public data repository and is published under the following DOI: 10.3929/ethz-b-000443651

## Appendix A: Crack patterns and analysis of crack kinematics

See Figs. 13, 14 and 15.
